# Caplacizumab in Lupus-Associated Thrombotic Thrombocytopenic Purpura (TTP): Navigating Autoimmunity and Microangiopathy

**DOI:** 10.7759/cureus.97906

**Published:** 2025-11-27

**Authors:** Arathi Kulkarni, Kiera Vaughn, Curtis Lacy, Tamin Sultani, Chandana Keshavamurthy

**Affiliations:** 1 Internal Medicine, University of Arizona College of Medicine - Phoenix, Phoenix, USA; 2 Hematology and Medical Oncology, University of Arizona College of Medicine - Phoenix, Phoenix, USA; 3 Radiology, University of Arizona College of Medicine - Phoenix, Phoenix, USA; 4 Rheumatology, University of Arizona College of Medicine - Phoenix, Phoenix, USA

**Keywords:** bleeding disorders and anemia, immune hemolytic anemia, immune ttp, systemic lupus erythematosus, ttp in sle

## Abstract

Thrombotic thrombocytopenic purpura (TTP) is a rare but potentially life-threatening complication associated with systemic lupus erythematosus (SLE). This condition is characterized by microangiopathic hemolytic anemia and thrombocytopenia, which can result from either an acquired deficiency of the ADAM metallopeptidase with thrombospondin type 1 motif 13 (ADAMTS13) or through complement-mediated mechanisms. We present the case of a 55-year-old female with a history of Sjögren's disease and a recent diagnosis of SLE. She exhibited severe anemia, thrombocytopenia, and symptoms suggestive of immune-mediated TTP with markedly reduced ADAMTS13 activity. Her diagnosis was confirmed through clinical evaluations, laboratory tests, and immunologic findings. After initially responding to urgent therapy with plasmapheresis, high-dose corticosteroids, and rituximab, the addition of caplacizumab led to a rapid and sustained improvement in her thrombocytopenia. This report highlights the successful use of caplacizumab in lupus-associated immune TTP and reviews diagnostic and therapeutic challenges.

## Introduction

Systemic lupus erythematosus (SLE) is an autoimmune disease affecting multiple organ systems. One rare but serious complication of SLE is immune-mediated thrombotic thrombocytopenic purpura (immune TTP), a known type of thrombotic microangiopathy (TMA) [1]. Immune TTP is characterized by a severe deficiency of the enzyme ADAM metallopeptidase with thrombospondin type 1 motif 13 (ADAMTS13) due to autoantibody-mediated inhibition [1,2]. This deficiency leads to the accumulation of large, highly adhesive von Willebrand factor multimers, which cause widespread platelet aggregation and activation, leading to hemolysis and damage to various organs [2].

TTP can present with severe neurologic manifestations caused by microvascular thrombosis, sometimes mimicking an acute stroke [2]. In those with severe symptoms, caplacizumab is often added as a therapy. Caplacizumab is a humanized bivalent variable domain-only immunoglobulin fragment, also known as a nanobody [3]. The drug specifically targets the A1 domain of von Willebrand factor, preventing its interaction with the platelet glycoprotein 1b-IX-V receptor and thereby inhibiting microvascular thrombosis [3]. By binding to von Willebrand factor and blocking its interaction with platelet-binding multimers, the drug rapidly increases platelet counts but predisposes to bleeding complications [2,3]. In a study published by Scully et al. (2019), a randomized clinical trial among patients with TTP, treatment with caplacizumab was associated with faster normalization of platelet count and a lower rate of TTP recurrence during the trial compared with placebo [3].

The overlap between SLE and TTP poses a diagnostic challenge, as both conditions can lead to cytopenias and hemolysis. This report highlights the need to recognize immune TTP as a potential complication in patients with SLE. Additionally, it emphasizes the importance of timely intervention and early use of available treatments, particularly caplacizumab, to optimize clinical outcomes.

## Case presentation

A 55-year-old female with a medical history of Sjögren's disease, noncompliant with hydroxychloroquine, was admitted to the hospital with progressive bruising, generalized weakness, dizziness, shortness of breath, and back and chest pain for one month. Her symptoms included dry eyes and mouth, as well as intermittent joint and bone pain, which she primarily experienced in cold weather. Although she had been prescribed hydroxychloroquine for her Sjögren's disease, she had not taken the medication. In the year leading up to her hospitalization, she reported only mild joint pain and occasional paresthesia in her hands, without any systemic symptoms, including fever, chills, weight loss, or loss of appetite. A thorough review of systems was negative for mucocutaneous ulcers, photosensitivity, alopecia, Raynaud's phenomenon, or neurological symptoms. She also had no history of thrombotic events, pregnancy loss, or prior renal disease.

Upon hospital admission, laboratory investigations revealed severe anemia and profound thrombocytopenia (Table 1). A peripheral blood smear demonstrated increased schistocytes, consistent with microangiopathic hemolytic anemia (MAHA). The reticulocyte count was elevated, and markers of hemolysis, including lactate dehydrogenase (LDH) and indirect bilirubin, were increased, while haptoglobin levels were undetectable. The direct Coombs test was negative for both immunoglobulin G (IgG) and complement component 3 (C3).

**Table 1:** Summary of laboratory investigations at presentation and follow-up. Laboratory findings at the time of hospital admission were consistent with microangiopathic hemolytic anemia (MAHA) and severe thrombocytopenia, supporting the diagnosis of thrombotic thrombocytopenic purpura (TTP) secondary to systemic lupus erythematosus (SLE). Up arrows (↑) indicate increased values, and down arrows (↓) indicate decreased values. anti-Sm: anti-Smith; anti-Sm/RNP: anti-Smith/ribonucleoprotein; anti-SSA: anti-Sjögren's-syndrome-related antigen A; anti-dsDNA: anti-double-stranded deoxyribonucleic acid; ANA: antinuclear antibody

Parameter	Initial Result	Follow-Up Result	Reference Range
Hemoglobin	6.6 g/dL (↓)	13.2 g/dL	12–16 g/dL
Hematocrit	19.7% (↓)	32.4%	36–46%
Platelet count	4 ×10³/µL (↓)	148 ×10³/µL	150–450 ×10³/µL
Peripheral blood smear	Schistocytes present	Resolved	None/rare
Reticulocyte count	7.8% (↑)	–	0.5–2.5%
Absolute reticulocyte index	4.04 (↑)	–	1.0–2.0
Lactate dehydrogenase (LDH)	640 U/L (↑)	206 U/L	140–280 U/L
Haptoglobin	Undetectable (↓)	77 mg/dL	30–200 mg/dL
Indirect bilirubin	Elevated (↑)	0.3 mg/dL	0.2–0.9 mg/dL
Direct Coombs test	Negative	–	Negative
ADAMTS13 activity	6% (↓)	–	>67%
ADAMTS13 inhibitor	Undetectable → Positive	Positive	Negative
D-dimer	1762 ng/mL (↑)	–	<500 ng/mL
Fibrinogen	Normal	–	200–400 mg/dL
Urinalysis	Protein + RBCs present (abnormal)	Normal	Negative
Complement C3	63 mg/dL (↓)	–	90–180 mg/dL
Complement C4	6 mg/dL (↓)	–	10–40 mg/dL
ANA	Positive	–	Negative
Anti-Sm, Sm/RNP, SSA, anti-chromatin	Positive	–	Negative
Anti-dsDNA	Negative	–	Negative
Antiphospholipid antibodies	Negative	–	Negative

Given the presence of severe thrombocytopenia and MAHA, ADAMTS13 activity was measured and found to be markedly decreased, confirming the diagnosis of TTP. An ADAMTS13 inhibitor, initially undetectable, was positive on repeat testing. The D-dimer level was elevated, while fibrinogen remained within normal limits. Urinalysis was initially abnormal with proteinuria and hematuria, which resolved on follow-up. Further immunologic evaluation revealed a positive antinuclear antibody (ANA) test and multiple extractable nuclear antigen (ENA) antibodies, including anti-Smith (anti-Sm), anti-Smith/ribonucleoprotein (anti-Sm/RNP), anti-Sjögren's-syndrome-related antigen A (anti-SSA), and anti-chromatin antibodies positive, along with hypocomplementemia (decreased complement components C3 and C4). Tests for anti-double-stranded deoxyribonucleic acid (anti-dsDNA) and antiphospholipid antibodies were negative.

**Figure 1:**
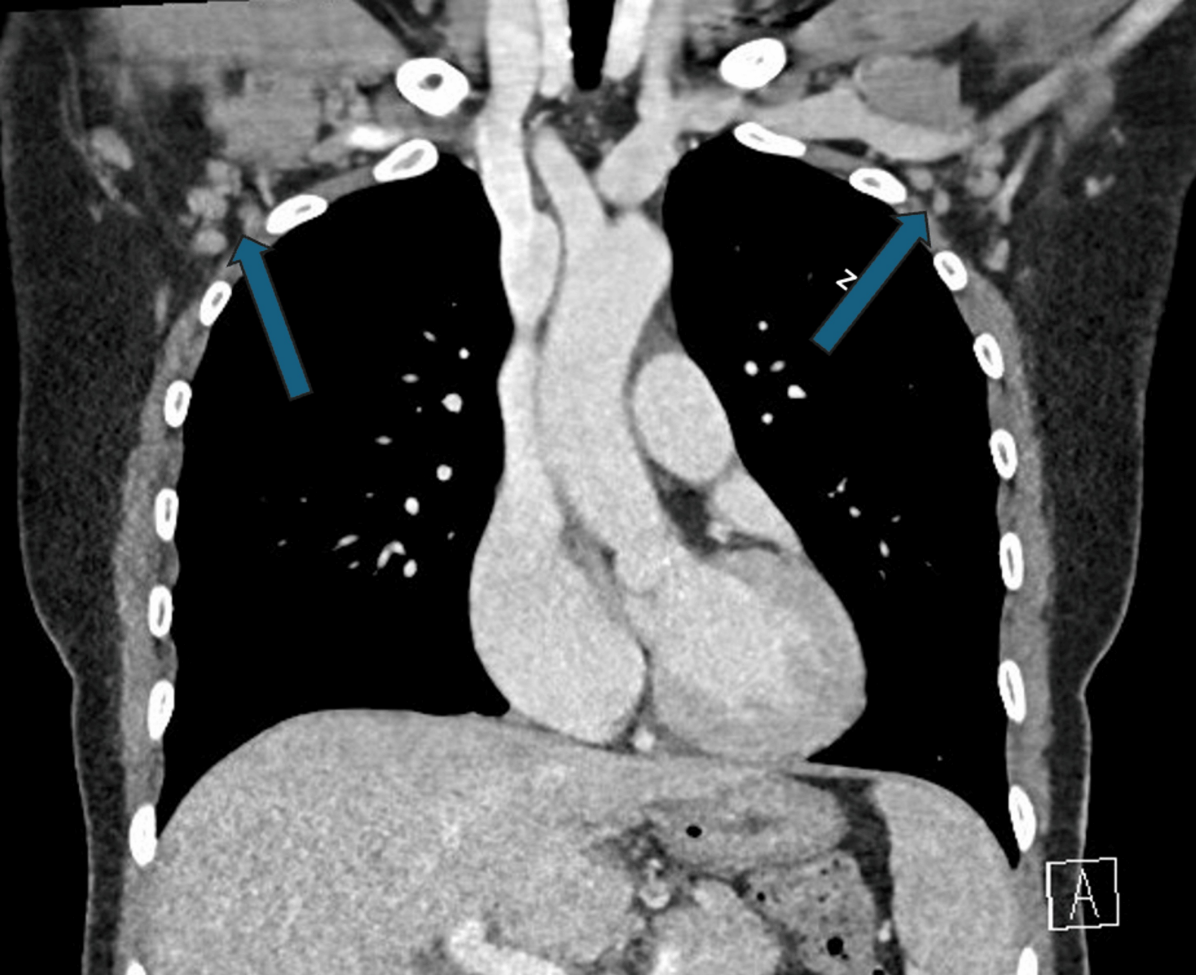
The arrows point to an increased number of lymph nodes in the axilla region, all measuring less than 1.5 cm.

**Figure 2:**
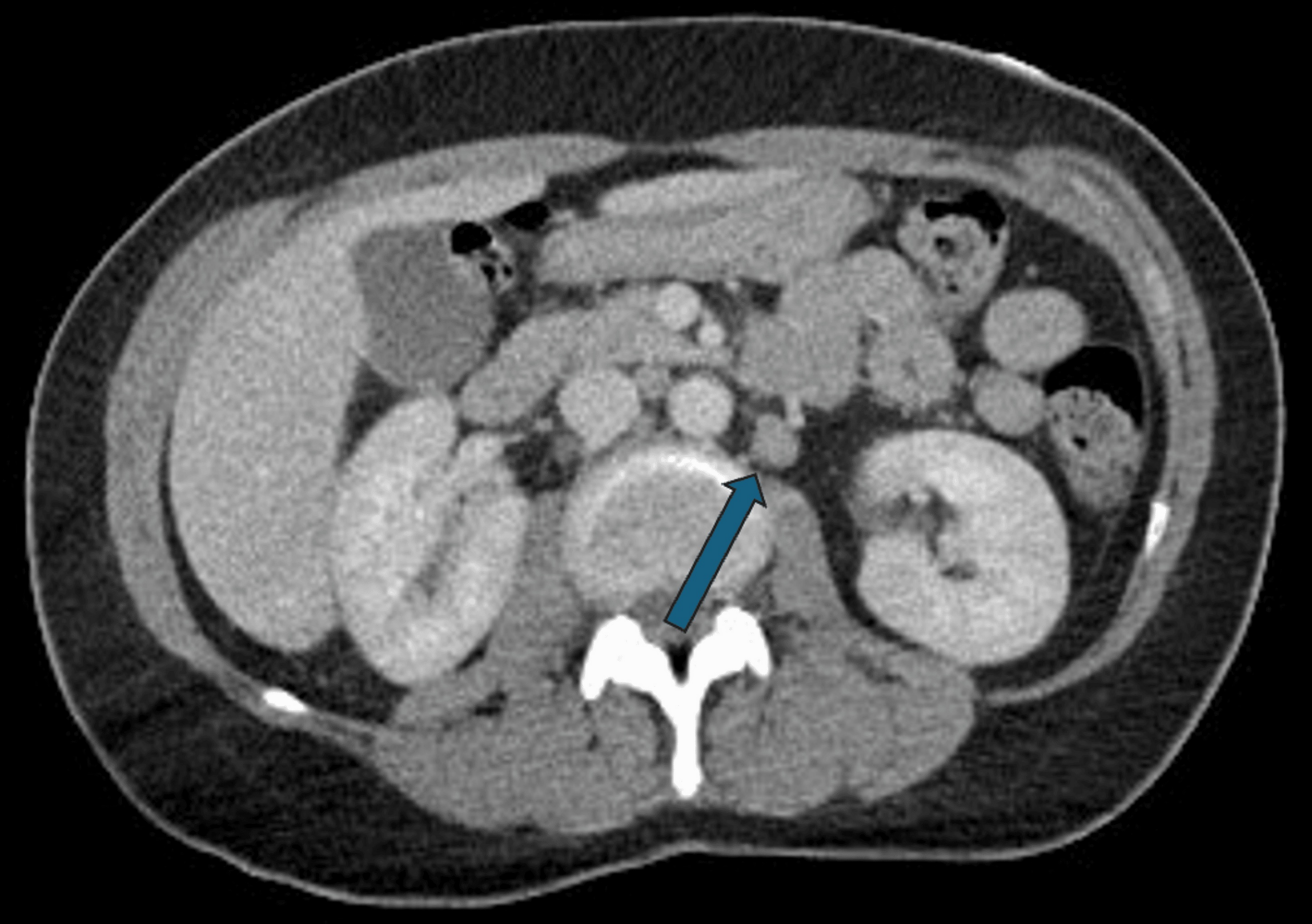
The arrow is pointing towards the lymph node in the retroperitoneal region, measuring less than 1 cm.

Computed tomography (CT) imaging revealed increased small lymph nodes in the axilla (Figure 1) and retroperitoneum (Figure 2), without significant organ involvement. Additionally, the infectious workup was negative for major viral and bacterial etiologies.

The overall findings fulfilled the American College of Rheumatology/European Alliance of Associations for Rheumatology (ACR/EULAR) classification criteria for SLE, with a Systemic Lupus Erythematosus Disease Activity Index 2000 (SLEDAI-2K) score of 3. Her platelet, lysis, active cancer, solid organ or hematopoietic stem cell transplant, mean corpuscular volume, international normalized ratio, and creatinine (PLASMIC) score of 5 indicated intermediate risk for TTP, and her French score of 3 signified high risk. With an ADAMTS13 activity <10% and the presence of an inhibitor, a diagnosis of acquired TTP secondary to SLE was established.

The patient was started on urgent therapeutic plasmapheresis (TPE) with fresh frozen plasma (FFP) for the removal of ADAMTS13 autoantibodies and to restore ADAMTS13 levels. She received corticosteroid therapy, beginning with 4 mg dexamethasone every six hours for three days, followed by 40 mg methylprednisolone every 12 hours for seven days. Due to refractory thrombocytopenia, she received an additional pulse of methylprednisolone (500 mg daily for three days) before transitioning to 60 mg prednisone daily.

Given the presence of an ADAMTS13 inhibitor and the diagnosis of systemic lupus, rituximab (375 mg/m² weekly for four weeks) was added following TPE sessions. This was intended to deplete B-cell populations responsible for autoantibody production, aiming to improve remission rates and reduce the likelihood of relapse. Hydroxychloroquine (200 mg daily, later increased to 300 mg daily) was also resumed as part of long-term management for lupus.

Despite the initial treatment, her platelet counts transiently improved before dropping again over the following weeks, leading to the addition of a 10 mg caplacizumab initial intravenous bolus and 10 mg subcutaneously daily for two months. Her hematological parameters improved significantly during her recent clinic visit, with a platelet count of 148 k/µL, normal hemoglobin of 13.2 g/dL, and hematocrit of 32.4%. Hemolysis labs normalized, with LDH at 206 U/L, haptoglobin at 77 mg/dL, and bilirubin at 0.3 mg/dL. Table 2 highlights the timing of medications and follow-up clinical laboratory results.

**Table 2:** This table outlines the timeline of therapies and medications administered during the patient's treatment course, including dosing details and corresponding clinical outcomes.

Phase/Timeline	Medication/Therapy	Dose & Schedule	Duration/Notes
Day 0 (initial presentation)	Therapeutic plasmapheresis (TPE) with fresh frozen plasma (FFP)	As per the TPE protocol	Urgent initiation
Day 0–3	Dexamethasone	4 mg every 6 hours	3 days
Day 4–10	Methylprednisolone	40 mg every 12 hours	7 days
Day 11–13	Methylprednisolone pulse	500 mg daily	3 days (for refractory thrombocytopenia)
Day 14 onward	Prednisone	60 mg daily	Maintenance phase following IV steroids
Following initial TPE sessions	Rituximab	375 mg/m² weekly	4 doses (weekly × 4 weeks)
Concurrent/long-term lupus management	Hydroxychloroquine	200 mg daily → increased to 300 mg	Chronic/ongoing
Several weeks later (after relapse of thrombocytopenia)	Caplacizumab	10 mg intravenous bolus -> 10 mg subcutaneously daily	2 months
Recent follow-up	-	-	Platelet count 148k/µL; hemoglobin 13.2 g/dL; hematocrit 32.4%; LDH 206 U/L; haptoglobin 77 mg/dL; bilirubin 0.3 mg/dL

Written informed consent was obtained from the patient for the publication of this case report.

## Discussion

Immune TTP is a microangiopathic process caused by ADAMTS13 inhibition, resulting in widespread platelet-rich thrombi, most prominently in the kidney and brain, and leading to organ ischemia and damage [2]. The formation of microthrombi is due to endothelial dysfunction, mediated by various mechanisms involving complement activation.

Insights from our case demonstrate longitudinal evaluation, including repeat ADAMTS13 testing and detailed immunologic profiling, which allowed for differentiation of immune TTP from other TMAs in a patient with overlapping autoimmune features. Additionally, the structured therapeutic timeline highlights the challenges of managing refractory disease and shows the benefit of multimodal therapy. Limitations of our case include the absence of complement genetic testing, which could have further clarified whether complement-mediated TMA contributed to the patient's presentation, particularly given her transient renal findings and hypocomplementemia. This gap reflects a broader diagnostic challenge in distinguishing immune TTP from complement-driven processes in complex autoimmune disease.

Conditions predisposing to TMA

TMA syndromes, which include TTP, often present with MAHA, schistocytes on peripheral blood smear in the setting of anemia, thrombocytopenia, abnormal LDH, haptoglobin, and indirect hyperbilirubinemia. However, some TMAs occur within the kidney [4]. It is unclear why there is a predilection for TMA in the vascular beds of specific organs, but it is likely associated with the vascular microenvironment related to genetic variables [1,4].

Among rheumatologic disorders associated with TMA, SLE appears to be the most frequently implicated, particularly in the setting of lupus nephritis [1]. Antiphospholipid antibody syndrome (APS) is also a well-recognized contributor to TMA. Systemic sclerosis-related TMA is less common and typically arises in the subset of patients who develop scleroderma renal crisis, while associations with Sjögren's syndrome, rheumatoid arthritis, and dermatomyositis are rare and largely limited to isolated case reports.

Rheumatologic diseases known to predispose patients to TMA include APS, catastrophic antiphospholipid syndrome (CAPS), systemic sclerosis, SLE, Sjögren's syndrome, vasculitis, nephritides, rheumatoid arthritis, and dermatomyositis [1]. Hemolytic uremic syndrome (ST-HUS) is caused by Shiga toxin-producing bacterial infections [5]. Malignancy, hypertension (HTN), organ transplants, medications, and infections such as *E. coli* have also been found to be risk factors [6]. Understanding the underlying mechanisms of TMA and its clinical manifestations is essential for timely diagnosis and management to prevent morbidity and mortality.

Clinical manifestations of TTP

The clinical manifestations of TTP are primarily the result of widespread microvascular thrombosis leading to MAHA, thrombocytopenia, and end-organ ischemia. Laboratory findings of MAHA include hemoglobin <10 g/dL, LDH greater than 1.5 times the upper limit of normal, undetectable serum haptoglobin, a negative direct erythrocyte antiglobulin test, and the presence of schistocytes on a peripheral smear [5]. Platelet abnormalities include thrombocytopenia with a platelet count <150 × 10⁹/L. End-organ damage predominantly affects the renal, cardiac, and nervous systems. Common symptoms include fatigue, fever, delirium, vision changes, and neurologic symptoms such as headaches, confusion, seizures, and stroke, as well as abdominal pain or nausea [2].

Congenital TTP

Primary TTP is caused by a deficiency of ADAMTS13, which is the result of a genetic mutation. ADAMTS13, also known as the von Willebrand factor (vWF)-cleaving protease, is responsible for cleaving ultra-large vWF multimers [3]. Deficiency in ADAMTS13 leads to an accumulation of ultra-large vWF multimers, which promote excessive platelet adhesion, aggregation, and activation, resulting in microthrombi formation [6].

Immune TTP

In immune TTP, ADAMTS13 deficiency is typically due to autoantibodies against the enzyme. It is important to note that more than 95% of TTP cases in adults are immune-mediated, resulting from autoantibodies against ADAMTS13 [2]. In patients with lupus, ADAMTS13 IgG antibodies inhibit the proteolytic activity of the metalloproteinase. These antibodies are present in 75% of immune TTP cases on initial testing, but many will become positive on subsequent testing [1,7,8].

In both congenital and immune TTP, the absence of ADAMTS13 leads to the accumulation of large multimers of vWF [6]. These multimers bind to platelets, leading to the formation of thrombi in the microvasculature. This can shear red blood cells and cause ischemic organ injuries, such as stroke and myocardial infarction.

TTP in systemic lupus

In patients with SLE, TTP is a potential complication. Lupus-associated TTP carries a significant risk of mortality, primarily from infections and renal injury. The condition presents a complex clinical picture, and clinicians may consider immune TTP and complement-mediated TMA as possible underlying mechanisms.

Patients with lupus are highly proficient at producing antibodies. Inhibitory antibodies targeting complement factor H and its regulatory proteins lead to primary complement-mediated TMA in these patients [9]. Complement overactivity is likely the underlying cause of TMA in lupus. Unchecked alternative pathway activation causes endothelial damage, MAHA, and end-organ injury [4,8]. In the literature, 0.5-1% of lupus patients have been reported to develop complement-mediated TMA [8]. In rare cases, complement-mediated TMA might be the presenting symptom of systemic lupus [1]. Complement-mediated TMA frequently involves the kidneys, often necessitating hemodialysis, at least temporarily, due to hypertensive emergencies [10]. Testing for genetic mutations and inhibitory antibodies against complement factor H and related proteins is essential [9]. Unfortunately, not all cases of complement-mediated TMA yield positive results on laboratory panels, as some cases may remain unidentified.

Evaluation and diagnosis

When evaluating for TTP in patients with systemic lupus, assessments include a complete blood count (CBC), a comprehensive metabolic panel, urinalysis with urine protein quantification, a reticulocyte count, LDH levels, haptoglobin measurement, a direct Coombs test, a coagulation profile, and a peripheral blood smear [1,4].

Common abnormalities observed include a platelet count of less than 30 x 10^9^/L. Patients often present with MAHA, characterized by hemolysis, elevated serum LDH, increased indirect bilirubin levels, a high reticulocyte count, low serum haptoglobin levels, and a negative Coombs test for anti-red cell antibodies and complement [2]. Schistocytes may be found at a frequency of 1% or greater among red blood cells [2]. Coagulation tests, such as prothrombin time and activated partial thromboplastin time, are typically normal. Additionally, acute kidney injury is noted in up to 50% of patients, with a rapid rise in creatinine levels to 1.5 mg/dL or higher [2,7].

An ADAMTS13 activity test and inhibitor level should be ordered, but given the three- to five-day turnaround time, treatment should begin empirically before results return when there is a high clinical suspicion. The diagnosis of immune TTP is confirmed by an ADAMTS13 activity level of less than 10% and ADAMTS13 IgG [2,11]. However, if acute TTP is suspected based on clinical and laboratory findings, treatment should not be delayed while awaiting ADAMTS13 results.

Patients with ADAMTS13 activity levels between 10% and 20% who are highly suspected of having immune TTP should undergo repeat ADAMTS13 activity testing to avoid missing a diagnosis [11,12]. The test should be conducted before TPE to prevent false elevations in donor plasma results [11].

To further confirm the diagnosis of immune TTP, patients should undergo anti-ADAMTS13 antibody testing. This can be done using a functional inhibitor assay/mixing study and/or an enzyme-linked immunosorbent assay (ELISA) for the detection of ADAMTS13 IgG antibodies [2,11]. Of note, 67% to 97.8% of patients with immune TTP have detectable ADAMTS13 antibodies; however, a small percentage may not have any detectable antibodies [2,7].

In patients with congenital TTP, ADAMTS13 IgG antibodies are not detected; instead, ADAMTS13 activity is typically below 10% [3,8,13]. In such cases, further genetic testing is necessary to identify a homozygous or compound heterozygous mutation in the *ADAMTS13* gene. No blood tests are currently available for immediate use in diagnosing complement-mediated TMA in systemic lupus [4].

The PLASMIC score, along with the French Thrombotic Microangiopathy Reference Center Score, can help distinguish and grade the severity of TTP from other TMAs [3,12]. A PLASMIC score of greater than 5 indicates a high risk of TTP [12]. Conversely, if the score is intermediate or low, it is important to consider alternative diagnoses. A systematic review of 13 validation cohort studies has demonstrated that a PLASMIC score of 5 or higher has a sensitivity of 99% and a specificity of 57% for diagnosing immune TTP. In patients with a score of 6 or more, treatment for TTP should be initiated without delay while ADAMTS13 levels are pending [12].

The French Thrombotic Microangiopathy Reference Center Score utilizes platelet count, serum creatinine levels, and the results of an ANA test. A score of 1 or higher indicates a sensitivity of 98.8% and a specificity of 48.1% for diagnosing immune TTP [12].

Treatment

Most of the early morbidity and mortality associated with acute immune TTP results from microvascular thrombosis, which can lead to end-organ ischemic injury. There are no standardized guidelines for managing immune TTP in patients with SLE. Data are extrapolated from TTP management in the general population. TTP is a medical emergency, and treatment should begin as soon as a presumptive diagnosis is made; delaying treatment with TPE can almost always be fatal [5]. If left untreated, TTP generally follows a natural course of progressive neurological decline, cardiac ischemia, irreversible kidney damage, and ultimately, death [5]. Common treatment modalities include glucocorticoids, immunosuppressive therapy, anticoagulation, antiplatelet agents, and TPE [12].

It is important to understand that even with the available treatment options, responses may be delayed, and the disease can progress quickly. This rapid progress can result in irreversible organ damage if not managed promptly. Therefore, early recognition, aggressive immunosuppression, and close monitoring of blood parameters and organ function are essential.

The 2025 focused update of the 2020 International Society on Thrombosis and Hemostasis (ISTH) guideline recommends that first-line treatment for immune TTP include a combination of TPE, corticosteroids, and medications such as rituximab and caplacizumab [11].

Plasmapheresis

If TTP is suspected, treatment should begin immediately with TPE while ADAMTS13 levels are pending, as this removes circulating inhibitory antibodies to ADAMTS13 and provides functional ADAMTS13 through donor plasma [11,14]. This treatment significantly reduces the mortality rate from 90% to between 9% and 20%. TPE must be continued daily until a platelet count response greater than 150 x 10^9^/L is achieved and sustained for two consecutive days. Risks associated with TPE include transfusion-related reactions, central line infections, thrombosis, bleeding, arterial injury, and bleeding from catheter insertion [15,16]. While plasmapheresis can be beneficial, it may not always be sufficient to reverse organ damage that has already occurred. There are data to support that TPE also works in complement-mediated TMA and has been shown to reduce kidney damage [1,4].

The role of plasmapheresis in lupus-related immune TTP is not well-defined; however, TPE is routinely offered to this population [1,8]. Historically, the management of TTP, when associated with autoimmune diseases like SLE, has focused on addressing the underlying immune dysregulation [1,7]. Initial treatment usually involves high-dose corticosteroids to reduce systemic inflammation, along with anticoagulation if there is evidence of thrombotic risk or confirmed thrombus formation [1,16].

Corticosteroids

Steroids reduce the production of autoantibodies against ADAMTS13 [7,16]. Two comparative observational studies have shown that combining corticosteroids with TPE leads to an absolute risk reduction compared to TPE alone [16]. A commonly used regimen is oral prednisone at a dose of 1 mg/kg body weight. In cases with severe neurological symptoms or elevated troponin levels, intravenous methylprednisolone at a dosage of 1000 mg daily for three days is recommended, followed by a prednisone equivalent of 1 mg/kg body weight, and continued throughout treatment until the ADAMTS13 activity increases to more than 10-20%, after which the dosage should be tapered to minimize toxicity [10].

Rituximab

B-cell depletion modulates the humoral immune response and plays a role in suppressing inhibitory autoantibodies to ADAMTS13 [17,18]. Rituximab, a monoclonal antibody that targets CD20 on the surface of B-cells, has been shown to reduce antibody production against ADAMTS13 [18]. This reduction helps restore ADAMTS13 levels and improves clinical outcomes in affected patients. However, there are currently no randomized controlled trials investigating the combination of rituximab with corticosteroids and TPE. Meta-analyses of six cohort studies have demonstrated that rituximab is effective in lowering the rate of clinical relapse when administered during an acute TTP episode [17-19]. Additionally, other anti-CD20 therapies, such as ofatumumab and obinutuzumab, can be utilized when rituximab is not a viable option [17,18].

Intravenous Immunoglobulin (IVIG)

IVIG is used in severe or refractory cases, as it may provide immunomodulatory effects [19]. These effects include neutralizing pathogenic antibodies, inhibiting complement activation, and modulating immune responses mediated by Fc receptors [19].

Caplacizumab

In 2019, caplacizumab was approved by the Food and Drug Administration (FDA) for adult patients with TTP, to be used in combination with TPE and immunosuppression treatments such as corticosteroids and rituximab [3]. Typical administration consists of a daily intravenous bolus followed by subcutaneous injections for 30 days post-TPE, continuing until ADAMTS13 activity is >20%. By inhibiting platelet incorporation into microthrombi, the drug reduces the occurrence of TTP-related deaths, disease recurrences, and other complications during the trial period compared with placebo [3].

TTP exacerbations can persist for up to 25 days after cessation of TPE treatment [3]. The activity of ADAMTS13 plays a crucial role in managing TTP, as relapses tend to occur in patients with severely suppressed ADAMTS13 activity, particularly those with levels below 10%. This highlights the potential benefit of monitoring ADAMTS13 activity to guide immunosuppressive treatment and determine whether to continue caplacizumab treatment beyond 30 days after TPE. Patients with severely suppressed ADAMTS13 activity (<10%) are at a higher risk of relapse, so monitoring these levels can help inform the duration of caplacizumab therapy. If ADAMTS13 activity remains low, extending treatment beyond the standard 30 days post-plasma exchange may be advantageous. Caplacizumab interferes with vWF, which is crucial for hemostasis, potentially leading to mucocutaneous bleeding similar to that in patients with von Willebrand disease [3]. Bleeding is generally mucocutaneous and manageable; dose interruption may be required for invasive procedures.

## Conclusions

This case illustrates the diagnostic complexity and management challenges of immune TTP secondary to SLE. Immune TTP in SLE is a rare but serious condition associated with significant morbidity and mortality. In this case, the diagnosis was confirmed by the presence of severe thrombocytopenia, schistocytes, and markedly low ADAMTS13 activity. It is essential to differentiate TTP from other hematologic disorders related to lupus, particularly CAPS and HUS. The absence of antiphospholipid antibodies ruled out CAPS, while the lack of renal dysfunction and absence of infection lowered the likelihood of HUS. The patient also did not have drug-induced hemolytic anemia, malignancy-associated TMA, or malignant HTN. Prompt recognition and early initiation of TPE, immunosuppressive therapy, and targeted agents, such as caplacizumab, are essential to improve outcomes. Long-term management should include a gradual taper of corticosteroids and selection of an appropriate maintenance immunosuppressant to prevent future SLE flares and TTP relapse. Early ADAMTS13 testing and timely incorporation of caplacizumab may help reduce mortality in autoimmune TTP, including lupus-associated cases.
